# Influence of Pro- and Anti-Inflammatory Cytokines in Th1 Polarization after Allogeneic Stimulation

**Published:** 2005-06

**Authors:** R. Silva, J. M. Morgado, A. Freitas, A. Couceiro, A. Orfao, F. Regateiro, A. Paiva

**Affiliations:** *Histocompatibility Centre of Coimbra, Portugal*

**Keywords:** flow cytometry, CCR5, Th1/Th2 polarization, GVHD, IL-18

## Abstract

The exogenous cytokine milieu can influence Th1/Th2 polarization. Besides the differential functional properties, T lymphocytes also acquire distinct profiles of chemokine receptors. Human Th1 lymphocytes preferentially express CCR5 and CXCR3 while Th2 lymphocytes express CCR3, CCR4 and CCR8. After their polarization into Th1 cells, grafted T lymphocytes mediate the development of graft-vs-host-disease, the major complication after bone marrow transplantation. We performed mixed lymphocyte cultures for ten days, with and without addition of IL-2, IL-4, IL-10, IL-12 and IL-18 at the third and sixth day of cultures. The expression of CXCR3 and CCR5, in CD4^+^ and CD8^+^ T lymphocytes was evaluated by flow cytometry, before and after ten days of culture. The exogenous addition of IL-2 or IL-12 favoured the Th1/Tc1 phenotype and IL-4 was also capable of inducing Th1 polarization. In opposition to IL-12, IL-18 didn’t induce a significant polarization into Th1 phenotype, an effect more similar to that induced by IL-10. This action could explain, at least in part, its possible protective effect in the incidence of acute and chronic graft-versus-host disease after allogeneic stem cell transplantation.

## INTRODUCTION

After activation, T lymphocytes acquire effector functions and can be subdivided, by a distinct cytokine production, into two subsets. T helper cells type 1 (Th1) predominantly produce INF-g, IL-2, TNF-b, IL-22 (1, 2) and IL-24 (3), and mediate a cellular immune response through the activation of macrophages and cytotoxic T cells. In contrast, Th2 cells predominantly secrete IL-3, IL-4, IL-5, IL-6, IL-9 and IL-10, potentiate the maturation of B cells and degranulation of mastocytes, thereby triggering a humoral immune response (4-11). Th1 and Th2 populations differentiate from naïve T cells after, at least, one round of antigen stimulation. Several mechanisms can influence Th1/Th2 polarization: the exogenous cytokine milieu, the nature of the peptide ligand, the activity of some costimulatory molecules and microenvironmentally secreted hormones (4, 6, 10, 11). It is actually accepted that Th1 cytokines inhibits the Th2 dominated immune response and vice versa. Such influence can be demonstrated by the anti-proliferative effect of INF-g on emerging Th2, and via inhibition of IL-4 and IL-5 dependent B lymphocyte differentiation. IL-4 inhibits Th1 cell development by down regulating the transcription factors promoting IFN-g synthesis (4-6).

Besides the differential functional properties, T lymphocytes also acquire different activation markers and distinct profiles of chemokines receptors (CKRs), that together with adhesion molecules (selectins and integrins) (12) modulate the migration and tissue homing of Th1 and Th2 to distinct peripheral sites of inflammation, where they can promote different types of inflammatory reactions (6, 8, 10, 13). Chemokines are chemoattractants, which direct T cells and other leukocytes into the inflammatory tissues (6, 14). Leukocytes respond to chemokines through specific G-protein-coupled receptors, the chemokine receptors, some of which are specific and interact with a single chemokine, whereas others are “shared” because they can bind multiple ligands (14, 16). There are two major groups for CKRs: CCR (1-10) that binds CC chemokines, and CXCR (1-5) binding CXC chemokines (17). Human Th1 and Th2 differentially express chemokine receptors, and therefore their recruitment is modulated in response to different chemokines (10, 18, 19). CCR5 and CXCR3 are preferentially expressed in human Th1 lymphocytes (Th1-associated CKRs), while Th2 lymphocytes preferentially express CCR3, CCR4 and CCR8 (Th2 associated CKRs) (9, 10, 18, 19); CXCR3 is also expressed by Th2 lymphocytes. Because of this different profile of expression, these receptors could be useful as markers of Th1/Th2 responses and tools to modulate polarized versions of T cell-dependent immunity (10).

It has been described (8) that the chemokine receptor expression on T cells is influenced by the activation state of the cells as by the cytokines present in the milieu, and correlates with distinct effector function. For example, while CXCR3 is expressed as a stable marker of memory Th1 and Th2 cells, CCR5 expression reflects the activation state of the cells, and it is up-regulated by IL-2 (8, 20). The expression of CCR5 and CXCR3 are closely linked; all T cells that express CCR5 also express CXCR3, which may thus be considered an “opportunistic Th1-associated marker” (18, 19, 21). This suggests that Th1 lymphocytes may be chemoattracted through either receptor.

Polarized T cells are involved in specific effector functions and are in progression of many diseases that also display strikingly polarized pathological features (21). It has been suggested that the balance between Th1/Th2 cytokines is largely determinate of the extent to which a cell-mediated immune response and a systemic inflammatory response develop after allogeneic bone marrow transplantation (BMT). The major complication after BMT is the development of graft-vs-host-disease (GVHD), which is mediated by grafted T lymphocytes after their polarization into Th1 cells (22-24). Therefore a cytokine capable of inducing a switch from Th1 to Th2 response inhibiting the production of IL-1 and TNF-a, may be a new possibility to take in account, with regard to the prevention and treatment of acute GVHD (22-24). In fact, it has been demonstrated that early administration of Th1 inducing cytokines, including IL-12, IFN-g and IL-2 have shown paradoxical ability to reduce the severity of acute-GVHD (25, 27). Some studies have failed to demonstrate beneficial effects to direct *in vivo* administration of Th2 cytokines in preventing or treating acute-GVHD (28, 29).

The aim of this study is to evaluate the influence of anti (IL-4, IL-10), and pro-inflammatory (IL-2, IL-12 and IL-18) cytokines in the Th1/Th2 polarization developed during an *in vitro* allogeneic response with peripheral blood (PB) mononuclear cells of healthy donors, in order to contribute to a possible development of novel therapeutic modalities and response to GVHD treatment. Since in cord blood transplantation (CBT) the incidence and severity of acute-GVHD seems to be reduced when compared with PB and BM (29, 34), we also performed our study in human cord blood samples.

## MATERIALS AND METHODS

### Blood samples

4 human cord blood samples were collected from healthy mothers at normal full term vaginal deliveries at the Bissaya Barreto Maternity Hospital (Coimbra, Portugal). The cord blood collections used for this study averaged 50 ml in volume and were collected to a heparinized container. 5 heparinized peripheral blood samples were obtained from adult healthy blood donors. Cord blood and adult peripheral blood mononuclear cells (CBMC and PBMC, respectively) were isolated by centrifugation over Ficoll-Hypac gradients (Lymphoprep™ Axis shield Pocas, Oslo, Norway). After the isolation, the cells were washed with Hanks Balanced Salts (HBSS -Gibco, Paisley, Scotland, UK) (15 min at 540g), and then resuspended in 1ml of RPMI-1640 medium (Gibco, Paisley, Scotland UK).

### Mixed Lymphocyte Cultures (MLC)

Cord blood mononuclear cells or peripheral blood mononuclear cells responder cells always presented several HLA mismatched class I and II with stimulator cells. Each sample of responder cells, at a concentration of 1 × 10^6^/ml, supplemented with 10% AB pooled human serum (Sigma, Saint Louis, MO, USA), was stimulated for 10 days in 96-well microtiter plates with 1 x 10^6^/ml allogeneic PBMC cells, treated with mitomycin C (Sigma, Saint Louis, MO, USA). Cell cultures (4 replicates for each studied cytokine and from each donor) were incubated at 37°C in a humidified atmosphere of 5% of CO_2_, and were feeded at the 3^rd^ and 6^th^ day, with rhIL-2 (40 U/ml) (Roche, Mannheim, Germany), rhIL-4 (200 U/ml) (Sigma, Saint Louis, MO, USA), rhIL-10 (100 U/ml) (PharMingen-BD, San Diego, C.A, USA), rhIL-12 (5 ng/ml) (R&D Systems, Europe), rhIL-18 (5 ng/ml), (MBL, Naka-ku Nagoya, Japan). Four replicates were performed without administration of exogenous cytokines.

### Chemokine receptors expression

At the 10^th^ day of culture two wells of each combination of the referred cultures were harvested and centrifuged for 5 minutes at 1500 rpm. The cells were stained for 15 minutes at room temperature in the dark with 10 μl of each of the specific anti-human MoAbs: CXCR3 FITC (clone 49801; R&D Systems, Europe), CCR5 PE (clone 2D7; Pharmingen-BD, San Diego, C.A., USA), CD8 PerCP (clone SK1; BD, San José C.A., USA) or CD4 PerCP (clone SK3; BD, San José C.A., USA) and CD3 APC (clone UCHT1; Pharmingen-BD, San Diego, C.A.,USA). After this incubation period, 2ml of FACS Lysing Solution (BDB) diluted 1:10 (v/v) in distilled water were added, and the samples were incubated for another 10 min, under identical conditions, in order to lyse non-nucleated red cells. Afterwards, cells were centrifuged (5 min, at 540 g) and the cell pellet was washed twice with 2 ml of phosphate-buffered saline (PBS-Dulbecco (1X) - Biochrom AG, Germany). Finally, cells were resuspended in 0.5 ml of PBS until analyzed in the flow cytometer.

### Flow cytometry data acquisition and analysis

Data acquisition was performed on a FACScalibur flow cytometer (BD, San José C.A., USA) equipped with the argon ion laser and a red diode laser. The number of events acquired for each sample was 10.000 on an electronic CD3^+^ gate, after a first acquisition of 10,000 of total events.

The identification of the different cell populations was made using “Paint-A-Gate 3.0.2 PPC” software program (BD, San José, USA). T lymphocytes were identified according to their positivity for CD3 and typical light scatter. Among them, CD4 or CD8 positive T cells were identified according to their reactivity with anti-CD4 or anti-CD8 monoclonal antibodies. The evaluation of the expression of these chemokines receptors was evaluated as the percentage of positive cells within each cell subset and their mean fluorescence intensity (MIF), expressed as linear fluorescence channels (arbitrary relative linear units scale from 0 to 10^4^).

### Statistical analysis

Statistical significance in the difference as observed in the results was assessed with SPSS 12.0 software using Mann-Whitney U-test or Wilcoxon signed-rank test, as appropriate.

## RESULTS

### Percentage of PB and CB T lymphocytes expressing CXCR3 and CCR5 before allogeneic stimulation

**Figure 1:**
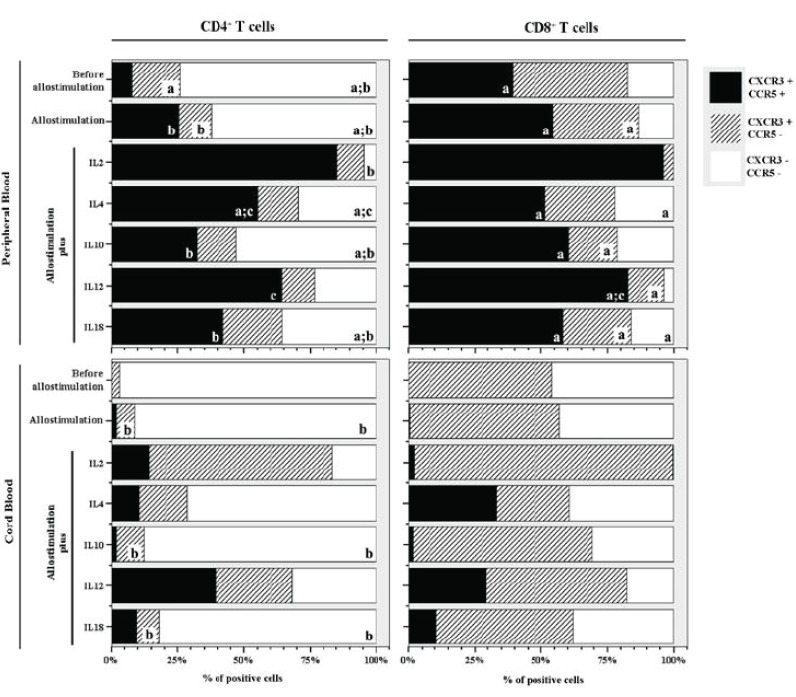
Expression of CXCR3 and CCR5 by CD8^+^ and CD4^+^ T cells from cord blood and adult peripheral blood before allogeneic stimulation and after allogeneic stimulation in the absence or in the presence of exogenous cytokines. Bars represent the median values of the percentage of positive cells. Statistically significant differences were considered when *P*<0.05. ^a^*P*<0.05 between PB and CB; ^b^*P*<0.05 between CD4^+^ and CD8^+^ T cells; ^c^*P*<0.05 when compared to “Allostimulation without cytokine”?

In PBMCs, our results show that CD4 T cells are mainly double negative for CXCR3 and CCR5 (68 (36-75)% vs 17 (13-37)% and 7.3 (0.0-25)% of CXCR3^+^/CCR5^-^and CXCR3^+^/CCR5^+^ respectively) while among CD8^+^ T cells higher percentages of CXCR3^+^/CCR5^-^ and CXCR3^+^/CCR5^+^ cells are present (42 (3.3-77)% and 39 (0-67)% respectively) (Fig. 1).

On CBMCs almost all CD4^+^ T cells are negative for CXCR3 and CCR5 (96(93-97)%) while CD8^+^T cells are, in similar proportions, double negative (46(5.1-98)%) or CXCR3^+^/CCR5^-^ (54(2.3-95)%). Among both populations of T cells no cells simultaneously expressing CXCR3 and CCR5 were present (Fig. 1). As expected, no CXCR3^-^/CCR5^+^ cells were found among PBMCs or CBMCs.

### Percentage of PB and CB T lymphocytes expressing CXCR3 and CCR5 after allogeneic activation

In PBMCs of healthy donors, our results show that the allogeneic stimulation, without exogenous addition of any cytokine, induced an increase in the percentage of CD4^+^ and CD8^+^ T cells co-expressing CCR5 and CXCR3 (22(15-51)% and 54(46-65)% of CD4^+^ and CD8^+^ T cells respectively) (Fig. 1).

The cytokines that induce, in a more significant way, the expression of CCR5 in both, CD4^+^ and CD8^+^, T lymphocytes are IL-2 (83 (67-88)% and 95 (87-98)% of CD4^+^ and CD8^+^ T cells respectively) and IL-12 (66 (20-82)% and 84 (63-87)% of CD4^+^ and CD8^+^ T cells respectively) (Fig. 1). However, in the CD4^+^ lymphocytes, the percentage of cells expressing CCR5 is also enhanced in the presence of IL-4 (49 (42-63)%) while the increases that IL-10 and IL-18 induced on CCR5 expression CXCR3 (34 (4.4-53)% and 45 (5.7-54)%) are considerably lower than that promoted by IL-2, IL-12 or IL-4 (Fig. 1).

With regard to CB T cells, flow-cytometric analysis showed that the allogeneic stimulus, in the absence of any cytokine didn’t induce a significant increase in the percentage of CD4^+^ and CD8^+^ T cells co-expressing CCR5 and CXCR3 (2.1 (0-19)% and 0.4 (0-17)% of CD4^+^ and CD8^+^ T cells respectively). IL-12 was the cytokine that induced, in a more significant way, the expression of CCR5 on the CD4^+^ subset of T lymphocytes (36 (18-61)%). IL-2, IL-4 and IL-18 also induced the increase of CCR5 expression (14 (7-29)%,11 (0-24)% and 9.4 (0-22)% respectively), but in a less significant way. Among CD8^+^ T cells from CB, the expression of CCR5 was enhanced by IL-4 (32 (0-41)%), IL-12 (27 (0-70)%) and IL-18 (11 (0-18)%) (Fig. 1).

## DISCUSSIONS

The Th1/Th2 polarization of T helper cell subsets may play an important role in the development of GVHD, a major obstacle to successful allogeneic hematopoietic stem cell transplantation. The immunopathophysiology of acute-GVHD is complex, and involves a “cytokine storm” amplified by the Th1 phenotype, which correlates with the development of acute GVHD. The inhibition of acute-GVHD can be achieved by a shift to Th2 polarization of donor T cells. Therefore a cytokine capable of inducing this switch in the donor T cells may be a new therapeutic agent, with regard to the prevention and treatment of acute-GVHD (22-24, 35). However, cytokines play a complex and dual role in GVHD, and can have either protectiveor deleterious effects.

In this study we saw that, in peripheral blood, there are CD4^+^ and CD8^+^ T cells that express CCR5 and, therefore, are already differentiated into Th1/Tc1 cells (Fig. 1).

After activation, T lymphocytes acquire effector functions and differentiate into Th1 or Th2 cells. In our work, the allogeneic stimulation of PBMCs induced an increase in the percentage of T cells co-expressing CXCR3 and CCR5, a Th1 phenotype.

Others have described (8, 17, 20) that the expression and responsiveness of certain chemokine receptors are up-regulated in T cells by stimulation with several cytokines such as IL-2 and IL-12. In line with the previous findings, after an allogeneic stimulation in the presence of IL-2 or IL-12, we detected an increase in the percentage of PB T cells that co-express CCR5 and CXCR3. Moreover, it seems that IL-4, a Th2 related cytokine, could also have the ability to mediate the up-regulation of CCR5 expression, at least in the CD4^+^ subpopulation of T cells, whereas IL-10, promoted a lower induction of CCR5 expression in our study. IL-10 was identified as a cytokine with a dual role; it has an important anti-inflammatory and immunosuppressive properties but, on the other hand, has immunostimulatory effects over B and T cells (36).

The exogenous addition of IL-18 caused a slight increase of CCR5 in CD4^+^ T cells.

From a functional point of view, IL-18 might be more related to IL-12 (35, 37-39) however the role of IL-18 in the differentiation of naive Th cells into Th1 cells is less clear. In the periphery, IL-18 synergistically induces the expression of the Th1 cytokines in the presence of IL-12 and Th2 cytokines in the presence of IL-2 (40, 41). IL-18 alone has minimal effect when compared to IL-18 in combination with IL-12, in inducing the Th1 cytokine IFN-γ production by tumor-draining lymph node cells in a murine model (41). In this regard in particular, our results show an effect of IL-18 more close to that of IL-10 than to that of IL-12. IL-18 levels correlate with GVHD course (42, 43). Despite reducing the severity of acute GVHD, preserves the GVL effect after bone-marrow transplantation (35, 44) and has the remarkable capacity to modulate acute GVHD when administered either to the donor or the recipient through distinct mechanisms (45).

The CCR5 positive cells are almost absent in CD4^+^ and CD8^+^ T cell subpopulations of CB. While in PBMCs IL-2 induced Th1 and Tc1 polarization in a similar way, in CBMCs the Tc1 polarization was less pronounced than Th1. Moreover, the allostimulation with the exogenous addition of IL-2 resulted preferentially in the expression of CXCR3 in the absence of CCR5 than in the co-expression of both receptors.

These results are in agreement with the naivity that characterizes CB cells and may, in part, explain the less severity of GVHD associated to cord blood transplantation.
